# Testing gender differences in psychological adjustment among early adolescents through a multigroup analysis of latent profiles

**DOI:** 10.1038/s41598-025-25420-7

**Published:** 2025-11-24

**Authors:** María Álvarez-Voces, Paula Villar, Karina Almón-Pazos, Estrella Romero

**Affiliations:** Underisk Group, Department of Clinical Psychology and Psychobiology, Faculty of Psychology, Institute of Psychology (IPsiUS), University of Santiago de Compostela, Santiago de Compostela, Spain

**Keywords:** Gender differences, Multigroup Analysis of Similarity, Latent profiles, Strengths and Difficulties Questionnaire, Psychological adjustment, Psychology, Human behaviour

## Abstract

Early adolescence is a crucial period for identifying psychological maladjustment and emerging gender differences. This longitudinal study explores gender differences in psychological maladjustment profiles among early adolescents, incorporating developmental predictors from earlier stages. The sample included 826 early adolescents (50% girls; *M*age = 10.72, *SD* = 0.71) who completed the Strengths and Difficulties Questionnaire–Self Report. A multigroup similarity in latent profile solutions was conducted to identify between-gender differences/similarities in latent psychological profiles, and multinomial logistic regressions were used to examine stable early predictors (i.e., parent-reported sociodemographic and temperamental factors) as well as more malleable parent-reported individual variables. Four profiles emerged: Moderately Externalizing, Internalizing, Comorbid, and Normative. Profile mean levels were similar across genders, but boys showed greater variability in prosocial behavior and peer problems. Girls were more likely to belong to the Internalizing and Normative profiles, whereas boys were more often found in the Externalizing and Comorbid ones. Key predictors (i.e., low socioeconomic status, low effortful control, high surgency/extraversion, and high emotional lability) showed similar effects across genders. These findings highlight the need for a nuanced understanding of gender differences in psychosocial adjustment and suggest that early temperament and emotional competences play a significant role in shaping adolescent mental health functioning.

**Supplementary Information:**

The online version contains supplementary material available at 10.1038/s41598-025-25420-7.

## Introduction

Early adolescence (ages 10–14) is a critical developmental stage when mental health challenges frequently arise. If these issues remain unaddressed, they can persist into adulthood, leading to long-term difficulties^1^. According to the World Health Organization^2^ approximately 14% of adolescents worldwide experience mental health disorders, which contribute to the global burden of disease, impair social and educational functioning, and impose significant economic costs on health and social care systems. Given this context, the early identification of subclinical symptoms becomes especially important, as timely monitoring and intervention may help prevent the escalation of problems and support healthier developmental outcomes^3–5^.

### Gender and patterns of mental health problems in adolescence

To address adolescent mental health effectively, it is vital to recognize the heterogeneity of psychological problems, and their varied manifestations. Mental health challenges rarely occur in isolation, highlighting the importance of identifying profiles and patterns that reveal how different difficulties co-occur and interact^6–9^. An important factor influencing these dynamics is gender, which plays a significant role in how adolescent mental health issues manifest. Research consistently shows that boys are more likely to exhibit externalizing behaviors such as conduct problems (CP), aggression, and hyperactivity, whereas girls tend to experience internalizing problems like anxiety, depression, and social withdrawal^10,11^. However, it remains unclear whether these problems manifest in similar profiles when they occur in the opposite gender (e.g., when girls display externalizing behaviors or boys internalizing ones). Similarly, uncertainty persists regarding whether the risk factors for various psychological challenges are consistent across genders or differ significantly, as findings in the literature remain inconclusive (e.g.,^12,13^).

#### Gender differences in risk factors for mental health problems

A variety of risk factors, including temperament and more malleable traits like emotion regulation, have been linked to internalizing and externalizing problems. Temperament, comprising biologically based emotional and behavioral tendencies emerging early in life^14^, has been associated with both internalizing and externalizing problems^15,16^. Three traits are particularly important: *effortful control*, a key aspect of self-regulation that involves attention and inhibitory control mechanisms; *surgency/extraversion*, characterized by impulsivity, high-intensity pleasure, high activity levels, and low shyness; and *negative affect*, akin to neuroticism, marked by heightened sadness, fear, anger, frustration, and discomfort, along with a reduced ability to recover from stress^17,18^. Boys generally exhibit lower effortful control and higher surgency/extraversion than girls, while negative affect levels are similar across genders^19,20^. Research indicates that the pathways connecting temperament to psychopathology might differ between genders^21,22^. In boys, lower effortful control and higher negative affect have been linked to externalizing problems, while in girls, these traits are more strongly associated with internalizing problems^23^. However, some studies find no significant gender differences in the relationship between effortful control and externalizing problems^24,25^. These results underscore the need for further exploration of the complex interactions between temperament, gender, and psychopathology^22^. Regarding more malleable variables, *emotion regulation*, a dynamic process that evolves throughout developmental stages and adapts to situational context^26^, has consistently been linked to both internalizing and externalizing problems^27^. This transdiagnostic factor plays a crucial role in mental health by involving processes that adjust the intensity and duration of emotions based on contextual demands^26^. Research suggests that gender influences emotion regulation strategies, with girls typically using more adaptive approaches, while boys are more likely to suppress emotions, potentially contributing to externalizing behaviors^28,29^. Further research is needed to understand these gender differences in emotion regulation and their contribution to gender-specific patterns of psychological problems in adolescence.

### Person-centered analysis of mental health problems in adolescence

In large-scale research on adolescent mental health, a range of robust instruments are available to assess key dimensions of psychological adjustment. Among these, the Strengths and Difficulties Questionnaire (SDQ;^30^ stands out for its widespread use and strong psychometric properties. The SDQ^30^ has been extensively used to examine the associations between risk and protective factors and both externalizing and internalizing problems^31^. However, most studies using the SDQ have relied on variable-centered approaches, which focus on relationships between variables across the entire sample. While valuable, this approach often fails to capture the heterogeneity of symptom patterns within individuals. In contrast, person-centered methods like Latent Profile Analysis (LPA) offer significant advantages. Specifically, they move beyond distinguishing between broad internalizing and externalizing problems categories, allowing for the identification of mixed or atypical constellations of symptoms that broadband scales may overlook. These refined profiles offer a more ecologically valid picture of adolescents’ adjustment and can guide tailored interventions that address co-occurring difficulties^32–34^. At the policy level, such insights can also inform prevention strategies and resource allocation that are responsive to the heterogeneity in youth psychological problems^35^. Despite its potential, person-centered methods remain underutilized. Additionally, while some studies have examined gender differences in this context, their focus has primarily been on the distribution of genders across profiles, with less emphasis on exploring how gender influences these patterns (e.g.,^36,37^). Gender could play a role in shaping how difficulties cluster together; for example, in boys, CP might co-occur with impulsive problems (e.g., hyperactivity), whereas in girls they might be more likely to appear alongside internalizing problems^38^.

### The current study

This study seeks to address critical gaps in understanding gender-specific patterns of psychological adjustment, by focusing on underlying risk and protective factors. It employs a multigroup analysis of similarity in latent profile solutions derived from SDQ data, providing a robust framework to assess the generalizability of these profiles across genders, thereby ensuring their relevance and applicability^39^. A key strength of this method is its ability to assess predictive similarity, which is crucial for translating person-centered findings into real-world applications. By identifying shared patterns, this analysis facilitates the development of a unified conceptual framework applicable across distinct groups. Conversely, observed differences between groups would highlight the need for tailored approaches to address gender-specific needs^40^. Thus, the specific aims of the study are: (1) examine gender differences in the number, structure, variance, and distribution of psychological adjustment profiles among early adolescents; and (2) investigate how stable early childhood factors (i.e., socioeconomic status [SES], and temperament), and malleable middle childhood factors (i.e., emotion regulation) are associated with these profiles according to gender. Longitudinal data from the ELISA study (Spain), collected at various stages of development, will be analyzed. Incorporating distal determinants will strengthen early prediction and identification of individuals at risk of maladjustment, while proximal factors will help identify key intervention areas during the critical period before adolescence. Consistent with previous research^6,9,41^, we hypothesize the emergence of distinct internalizing and externalizing problems groups, along with a co-occurring group. We further expect that girls will be more likely than boys to belong to the internalizing group, whereas boys will be more likely to belong to the externalizing problems group than girls^2,10^. Given the inconsistencies in prior studies, no assumptions will be made regarding the specific gender-related relationships between risk factors and psychological adjustment profiles.

## Methods

### Participants

The present study utilized data from children who participated in the longitudinal ELISA Project (*Estudio Longitudinal para una Infancia Saludable*), conducted in Galicia (NW Spain). The ELISA project started in 2017 and, as of 2024, encompasses seven data collections (*n* = 2471 children; 48.1% girls). The final sample utilized in this study was composed of 826 children (50% girls) who responded to the SDQ-SR^30^ in 2023, with their primary caregivers providing data in 2018 and 2022 (88.1% mothers, 11.8% fathers and 0.1% others). Three waves were established for the purposes of this study: T1 (2018; age *M* = 5.62, *SD* = 0.75, *range* = 4–7) and T2 (2022; age *M* = 9.63, *SD* = 0.74, *range* = 8–11) for parent-reported predictors, and T3 (2023; age *M* = 10.72, *SD* = 0.71, *range* = 10–12) for the identification of psychological adjustment profiles with children-reported information. The number of participants in T3 (*n* = 826) was slightly higher than in T1 (*n* = 772) and T2 (*n* = 719) due to the study’s longitudinal design, which allows re-engagement of temporarily disengaged participants. Comparisons between children who missed a data collection and those who completed all revealed no statistically significant differences in age (*F*(2, 823) = 2.86, *p* = 0.058), gender (χ^2^(1) = 3.11, *p* = 0.211), emotional symptoms (*F*(2, 823) = 2.86, *p* = 0.079), CP (*F*(2, 823) = 2.32, *p* = 0.099), hyperactivity/inattention (*F*(2, 823) = 2.44, *p* = 0.088), peer relationship problems (*F*(2, 823) = 2.97, *p* = 0.052), or prosocial behavior (*F*(2, 823) = 0.48, *p* = 0.622). However, differences in socioeconomic status (SES) were found (*F*(2, 798) = 1.62,* p* = 0.022; = η^2^ = 0.01), and participants who completed all data collections exhibited higher scores than those who missed a data collection (*p* = 0.020). This pattern aligns with findings from prior longitudinal research^42^, though the effect size was small. Most of the children were Spanish (≈95%). At the first data collection of this study, 83.8% of mothers and 94.4% of fathers worked outside the home. In terms of parent’s education, 10.3% of mothers and 26.1% of fathers had attained the highest level of compulsory school education, 9.6% and 8.9% had completed a higher level of non-compulsory school education, 26.4% and 28.9% had completed vocational training studies, 44.8% and 29.6% had university studies and 8.7% and 5.8% had postgraduate studies.

### Measures

#### For profiling (T3)

The SDQ-SR^30^ was utilized to assess psychological adjustment in early adolescents. The SDQ’s content and structure align with the primary categories of mental disorders outlined in the Diagnostic and Statistical Manual of Mental Disorders [DSM-IV]^43^, serving as a screening tool to identify potential psychological issues in community samples^44^. The Spanish version of the SDQ has demonstrated strong psychometric properties, with good reliability across its subscales (ordinal α = 0.71–0.75) and evidence of construct validity, including support for the five-factor structure and measurement invariance across gender and age^45^. The SDQ consists of 25 items, divided into five subscales, each containing five items: emotional symptoms (e.g., “I worry a lot”; ordinal α = 0.78), CP (e.g., “I fight a lot. I can make others do what I want”; ordinal α = 0.70), hyperactivity/inattention (e.g., “I finish the work I’m doing. my attention is good”; ordinal α = 0.77), peer relationship problems (e.g., “other children or young people pick on me or bully me”; ordinal α = 0.61), and prosocial behavior (e.g., “I am kind to younger children”; ordinal α = 0.75). The response scale is a 3-point Likert scale (0 = not true to 2 = certainly true). For profiling purposes, scores from the SDQ subscales were treated as continuous variables and standardized into *Z*-scores. This approach is supported by studies suggesting that SDQ scores should be treated as continuous measures, as they provide valuable information across the scale and at the subscale level^46^. In line with prior research, we followed the broader structure whereby emotional symptoms and peer problems are conceptualized jointly as internalizing problems, CP and hyperactivity/inattention as externalizing problems, while prosocial behavior is retained as an independent subscale^47^. This structure has shown good validity in community samples and guided how we defined the adjustment profiles in this study.

#### For predicting profile membership (T1 and T2)

SES was assessed using ad hoc items in T1 (α = 0.66), which measured parental education level (1 = without basic studies to 6 = postgraduate degree), family income (1 = serious problems making ends meet to 4 = well off), and family financial situation in meeting daily expenses (1 = never worried to 5 = worried every day). A composite SES score was calculated by averaging the *Z*-transformed values of those variables. This measure has been used in prior research and demonstrated psychometric adequacy^48^.

The Children’s Behaviour Questionnaire Very Short Form [CBQ-VSF]^17^ was used to assess temperamental traits in T1. Specifically, the Spanish version, translated by the Research Group in Child Psychology (GIPSE) from the University of Murcia (Spain) was employed. This questionnaire consists of 36 items rated on a 7-point Likert (1 = totally false to 7 = totally true). The CBQ-VSF consists of three subscales: effortful control (12 items; “When drawing or coloring in a book, shows strong concentration”; α = 0.66), surgency/extraversion (12 items; “Is full of energy, even in the evening”; α = 0.68), and negative affect (12 items; “When angry about something, she/he tends to stay upset for ten minutes or longer”; α = 0.73).

The Emotion Regulation Checklist [ERC]^49^ was used to measure emotion regulation and lability/negativity T2. This questionnaire consists of 24 items rated on a 4-point Likert scale (1 = never to 4 = almost always). The ERC includes two subscales: emotion regulation (eight items; “Displays appropriate negative emotion (for example, anger, fear, frustration, distress) in response to hostile, aggressive, or intrusive acts by peers”; α = 0.72) and lability/negativity (16 items; “Is easily frustrated”; α = 0.82).

### Procedure

A total of 126 public, charter, and private schools in Galicia were initially contacted, of which 72 agreed to participate. Within these schools, 25–50% of families consented to participate. The questionnaires were distributed to one of the primary caregivers, mostly mothers, and to the children in 2023. Teachers supervised the distribution and collection of the questionnaires from families, while the research team administered the questionnaires directly to the children in the schools. We obtained informed consent in advance from each child’s primary caregiver. Confidentiality was maintained through pseudo-anonymization by assigning each participant a unique ID and providing an alphanumeric code to securely access the questionnaire. While no monetary rewards were provided, parents received personalized reports with recommendations based on their child’s questionnaire results. Additionally, formative discussions for teachers and families were available upon request throughout the study. We standardized the questionnaire administration process as much as possible, despite the diversity of schools. The research study and procedures were reviewed and approved by the Bioethics Committee at the University of Santiago de Compostela (Reference: USC-21/2020. Approval Dates: June 17, 2016, and November 9, 2020). All procedures adhered to the guidelines and regulations established by the approving institutions, ensuring compliance with the Declaration of Helsinki and relevant ethical codes.

### Statistical analyses

Analyses were conducted using MPlus 7^50^ and IBM SPSS Statistics 29. Descriptive statistics were calculated for the full sample, and mean comparisons were used to explore gender differences in the variables of interest. LPAs and multigroup similarity analyses were conducted to identify latent profiles and assess gender-based similarities. Finally, Multinomial Logistic Regression (MLR) was employed to estimate the associations between predictors and membership in the SDQ profiles.

#### Separate latent profile analyses

To assess whether the same number of profiles could be estimated for both genders (a prerequisite for further multigroup analysis), separate LPAs were conducted for boys and girls using the five SDQ subscales (i.e., emotional problems, CP, hyperactivity/inattention, peer relationship problems, and prosocial behavior) as indicators. Analyses were conducted using the Maximum Likelihood Robust (MLR) estimator and missing data were handled using the Full Information Maximum Likelihood (FIML) method. FIML has been shown to yield unbiased parameters, particularly when comparing to listwise deletion, and is especially effective in addressing random data loss and higher rates of missing data^51^. Models of one to five profiles were estimated and the best-fitting model was determined using criteria established in previous studies^52–54^, including: 1) lower Akaike Information Criterion [AIC], Bayesian Information Criterion [BIC], and Sample-Size Adjusted BIC [SSABIC]; 2) statistically significant Lo-Mendell-Rubin [LMR], Lo-Mendell-Rubin adjusted [LMRt], and Bootstrapped Likelihood Ratio Test [BLRT]; and 3) theoretical interpretability of profiles. The selection of the number of latent profiles can be influenced by sample size, as significance tests tend to favor more complex models with larger samples^55^. As a result, fit indices may continue to improve with the addition of more profiles without reaching a clear minimum. To address this, elbow plots are often used to graphically represent the gains associated with additional profiles. The point at which the slope flattens indicates the optimal number of profiles in the data^56^. In cases of conflicting fit indices, priority was given to theoretical interpretability^57^, the significance of the BLRT, and the lowest values of BIC and SSABIC^53^. Entropy values were also reported to indicate the precision of profile classification (ranging from 0 to 1); however, entropy should not be used as a criterion for determining the optimal number of profiles^58^. In the initial models, both the means and variances of the five SDQ variables were freely estimated across all profiles and in both samples. However, repeated estimation attempts failed to achieve convergence, suggesting that the models may have been over-parameterized^59^. To resolve this, we followed established recommendations in the LPA literature^60^ and re-estimated the models using freely estimated means while fixing the variances. This approach resulted in successful convergence and yielded a stable, well-replicated solution across both samples.

#### Multigroup analysis of similarity

For multigroup analysis of similarity, we followed the six-step process outlined by Morin et al.^40^ which includes configurational, structural, dispersion, distributional, predictive, and explanatory similarity. This comprehensive approach allows for a detailed examination of both the profiles and their variations across gender groups. To this end, the KNOWNCLASS function was used to specify the gender groups. As in the initial models, convergence issues also arose in the multigroup framework, and the same approach described above (i.e., fixed variances of the five SDQ variables across profiles) was applied to achieve stable solutions.

*Configural Similarity.* The first step in the process is to determine whether the same number of profiles can be identified across groups, a condition referred to as configural similarity. This is evaluated through separate LPAs for each group (see the subsection below). Once this condition is established, a multiple group model can be estimated, allowing equality constraints to be introduced progressively. In our case, the optimal solution for both boys and girls included four profiles, which made it possible to establish a joint configural model for subsequent comparisons.

*Structural, Dispersion, and Distributional Similarity.* The models for structural, dispersion, and distributional similarity were estimated as follows: 1) in the structural model, equality constraints were imposed across genders on the within-profile means of the variables; 2) in the dispersion model, equality constraints were imposed across genders on both the within-profile means and variances of the variables; and 3) in the distributional model, equality constraints were imposed across genders on the within-profile means, the variances of the variables and the relative profile size. Model fit indices (AIC, BIC, SSABIC) were compared to determining the best-fitting model.

*Predictive Similarity.* Predictors were added to the model to determine if their effects were consistent across genders. The initial values from the best-fit model (i.e., the structural similarity model) were used. Two models were run for each set of predictors: one where the effects of the predictors were estimated freely across genders, and another where the effects of the predictors were constrained to be the same for both genders.

## Results

Tables S1 to S3 in the Supplementary Material present the correlations between the studied variables for the full sample, as well as separately for boys and girls. Table 1 presents descriptive statistics and gender differences in the variables assessed. For the profiling variables, findings indicate that boys scored significantly higher than girls in CP, hyperactivity/inattention, and peer relationship problems, whereas girls scored higher than boys in emotional symptoms and prosocial behavior, with small effect sizes. Regarding predictors, no significant differences were found in SES. For temperamental predictors, boys had higher levels of surgency/extraversion, while girls scored higher in negative affect and effortful control, with small effect sizes overall, except for effortful control, which showed a large effect size favoring girls. Regarding emotional predictors, girls scored higher in emotion regulation, while boys showed greater emotional lability and negativity, although the effect sizes were small.

**Table 1:** Descriptive statistics and gender differences in the variables studied.

**Measures**	**Scale range**	**Global**			**Boys**			**Girls**			*t* (*df*)	Cohen’s* d*
		*n*	Mean (*SD*)	Median	*n*	Mean (*SD*)	Median	*n*	Mean (*SD*)	Median		
For profiling (T3)												
Emotional symptoms^a^	0–2	826	0.63 (0.48)	0.60	413	0.55 (0.45)	0.60	413	0.71 (0.50)	0.60	− 4.76 (814.35)***	− 0.33 (small)
Conduct problems^a^	0–2	826	0.43 (0.35)	0.40	413	0.49 (0.38)	0.40	413	0.36 (0.31)	0.40	5.46 (793.55)***	0.38 (small)
Hyperactivity/Inattention	0–2	826	0.83 (0.49)	0.80	413	0.88 (0.49)	0.80	413	0.78 (0.49)	0.80	3.14 (824)**	0.22 (small)
Peer relationship problems	0–2	826	0.38 (0.33)	0.40	413	0.42 (0.33)	0.40	413	0.34 (0.32)	0.20	3.66 (824)***	0.26 (small)
Prosocial behavior^a^	0–2	826	1.72 (0.30)	1.80	413	1.66 (0.33)	1.80	413	1.78 (0.25)	1.80	− 5.93 (824)***	− 0.41 (medium)
For predicting												
Socioeconomic status	*Na*	801	0.09 (0.66)	0.14	401	0.10 (0.64)	0.15	400	0.07 (0.66)	0.11	0.60 (99)	
Effortful control^a^ (T1)	1–7	768	5.13 (0.76)	5.17	381	4.91 (0.77)	4.92	387	5.34 (0.68)	5.33	− 8.14 (751.01)***	− 0.59 (medium)
Surgency/Extraversion^a^ (T1)	1–7	769	4.42 (0.86)	4.42	382	4.49 (0.87)	4.50	387	4.35 (0.84)	4.42	2.36 (765.27)**	0.17 (small)
Negative affect (T1)	1–7	768	4.08 (0.93)	4.08	381	3.97 (0.94)	4	387	4.20 (0.91)	4.25	− 3.43 (766)***	− 0.25 (small)
Emotion regulation^a^ (T2)	1–4	718	3.50 (0.41)	3.63	356	3.44 (0.44)	3.50	362	3.55 (0.36)	3.63	− 3.54 (688.13)***	− 0.27 (small)
Lability/Negativity^a^ (T2)	1–4	718	1.62 (0.35)	1.56	356	1.66 (0.39)	1.63	362	1.58 (0.29)	1.56	3.39 (662.62)***	0.25 (small)

^a^Welch t-test is reported because Leven’s test is significant (*p* < 0.05), suggesting a violation of the assumption of equal variances; **p < 0.01; ***p < 0.001. Effect size (Cohen’s *d*): 0.2 = small; 0.5 = medium; 0.8 = large^61^.

### Cross-gender similarity in SDQ profiles

Table 2 presents the fit indices of the LPAs conducted separately by gender, as well as the results from the multigroup analysis of similarity. For girls, the four-profile solution was selected based on its lower AIC, BIC and SSABIC values relative to models with fewer number of profiles and a lower BIC compared to the five-profile model, and due to its theoretical significance. For boys, the four-profile solution was chosen due to its lower BIC compared to models with fewer profiles, along with clearer theoretical interpretability than the five-profile solution. The four-profile model yielded distinct, meaningful profiles, while the five-profile solution identified two profiles that were both normative, showing low levels across emotional symptoms, CP, hyperactivity/inattention, peer relationship problems, with average prosocial behavior. In addition, to complement the information, we examined elbow plots of the information criteria (see Figure S1 in the Supplementary Material). These plots showed that the values of nearly all indices decreased and began to plateau at four profiles, suggesting this as the optimal solution. Given that the four-profile solution emerged as optimal for both genders, a multigroup model with four profiles was estimated (i.e., configural model). Subsequently, structural, dispersion, and distributional models were estimated and compared. The structural similarity model was retained for the following steps due to the lower values of AIC and SSABIC and the almost identical value of BIC. As described in the Method section, variances were fixed across profiles due to convergence issues. This model also demonstrated strong classification accuracy with an entropy of 0.86. The mean posterior probabilities of membership for the primary profiles ranged from 0.75 to 0.91 for boys and from 0.79 to 0.94 for girls, indicating reliable profile classification across both genders (see Table S4 in the Supplementary Material for details).

**Table 2:** Fit Results From LPA For Girls and Boys and Multigroup Analysis of Similarity.

Model	*k*	AIC	BIC	SSABIC	Entropy	SP	LMR	LMRt	BLRT
							*p* value		
Profile enumeration: Girls									
One profile	1	5624.73	5664.97	5633.23	–	–	–	–	–
Two profiles	2	5318	5382.38	5331.61	0.88	17.3%	0.001	0.001	< 0.001
Three profiles	3	5236.09	5324.61	5254.79	0.77	12.4%	0.103	0.109	< 0.001
** Four profiles**	**4**	**5190.68**	**5303.34**	**5214.49**	**0.84**	**5%**	**0.058**	**0.061**	** < 0.001**
Five profiles	5	5169.44	5306.23	5198.35	0.79	5.5%	0.499	0.507	< 0.001
Profile enumeration: Boys									
One profile	1	5971.21	6011.44	5979.71	–	–	–	–	–
Two profiles	2	5678.12	5742.50	5691.73	0.77	32.2%	< 0.001	< 0.001	< 0.001
Three profiles	3	5622.63	5711.15	5641.34	0.69	20.2%	0.034	0.037	< 0.001
** Four profiles**	**4**	**5575.77**	**5688.42**	**5599.57**	**0.76**	**9.8%**	**0.056**	**0.060**	** < 0.001**
Five profiles	5	5542.73	5679.52	5571.63	0.80	1.7%	0.372	0.382	< 0.001
Cross-gender similarity									
Configural	4	11,913.53	12,182.37	12,001.36	0.87	9%	–	–	–
**Structural**	**4**	**11,996.37**	**12,170.88**	**12,053.38**	**0.86**	**8.1%**	**–**	**–**	**–**
Dispersion	4	12,018.46	12,169.39	12,067.77	0.85	6.6%	–	–	–
Distributional	4	12,054.41	12,191.19	12,099.10	0.85	7.9%	–	–	–
Predictive similarity									
SES									
Freely estimated across genders	4	11,551.13	11,612.04	11,570.76	0.86	–	–	–	–
** Equality estimated across genders**	**4**	**11,553.70**	**11,600.56**	**11,566.80**	**0.86**	**–**	**–**	**–**	**–**
Temperament									
Freely estimated across genders	4	11,057.13	11,173.23	11,093.84	0.86	–	–	–	–
** Equality estimated across genders**	**4**	**11,042.73**	**11,117.03**	**11,066.22**	**0.86**	**–**	**–**	**–**	**–**
Emotional variables									
Freely estimated across genders	4	10,279.36	10,366.32	10,305.99	0.87	–	–	–	–
** Equality estimated across genders**	**4**	**10,277.42**	**10,336.91**	**10,295.63**	**0.86**	**–**	**–**	**–**	**–**

Bold indicates the final solution.* k* = number of profiles; AIC = Akaike Information Criterion; BIC = Bayesian Information Criterion; SSABIC = Sample-size adjusted BIC; SP = Smallest profile; LMR = Lo, Mendell and Rubin likelihood ratio test; LMRt = LMR adjusted; BLRT = Bootstrap Likelihood Ratio Test.

### Profile description

**Fig. 1:**
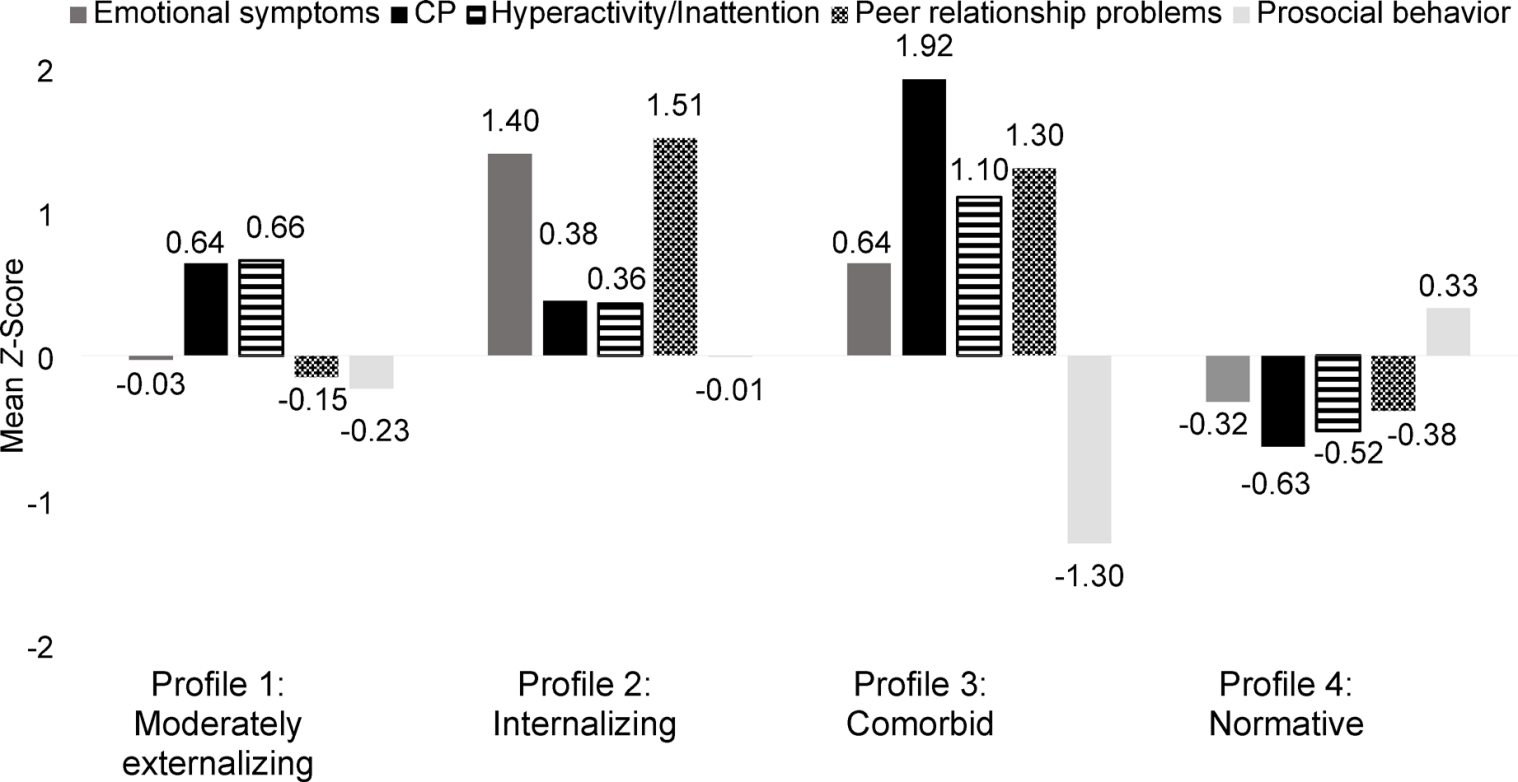
Final 4-profile Solutions of the Structural Similarity Model.

Figure 1 shows the *Z*-means for the compositional variables of the four profiles in the structural similarity model. Profile 1 (≈25.9%), exhibited moderate CP and hyperactivity/inattention scores, with average scores on emotional symptoms, peer relationship problems, and prosocial behavior. As a result, it was named the *Moderately Externalizing* profile. Profile 2 (*Internalizing*; ≈9.2%) showed high scores in emotional symptoms and peer relationship problems, alongside average scores on CP, hyperactivity/inattention, and prosocial behavior. Profile 3 (*Comorbid*; ≈8%) was characterized by very high scores in CP, hyperactivity/inattention, peer relationship problems, along with moderate emotional symptoms and notably low prosocial behavior. Also, it is characterized by very low scores on prosocial behavior. Finally, Profile 4 (*Normative*; *≈*56.9%) was the largest profile and showed average scores on all variables. Differences between profiles in the compositional variables were supported by non-overlapping confidence intervals in most cases. Exceptions were observed in prosocial behavior, hyperactivity/inattention, and CP between the *Moderately Externalizing* and *Internalizing* profiles; in peer relationship problems between the *Internalizing* and *Comorbid* profiles; and in emotional problems between the *Moderately Externalizing* and *Normative* profiles (see Table S5 for detailed means and confidence intervals of the compositional variables).

Dispersion and distribution similarities across genders were not supported, indicating that the variances of the compositional variables and the distribution of early adolescents within each profile varied across genders. Regarding dispersion, boys displayed greater variability in prosocial behavior and in peer relationship problems, as evidenced by the non-overlapping confidence intervals for variance estimates between genders (see Table 3). In terms of distribution, based on estimated posterior probabilities the *Normative* (48.1% vs 65.7%; χ^2^(1) = 25.96, *p* < 0.001; φ = 0.18) and *Internalizing* (6% vs 12.3%; χ^2^(1) = 9.68, *p* = 0.002; φ = 0.11) profiles were more frequent in girls, whereas the *Moderately Externalizing* (32.5% vs 19.3% ; χ^2^(1) = 18.85, *p* < 0.001; φ = − 0.15) and *Comorbid* (13.3% vs 2.8%); χ^2^(1) = 31.18, *p* < 0.001; φ = − 0.19) profiles were more frequent among boys.

**Table 3:** Variance Estimates of the Multigroup 4-Profile Structural Model for Boys and Girls.

Domain	Boys			Girls		
	Estimate	Lower CI	Upper CI	Estimate	Lower CI	Upper CI
Emotional problems	0.67	0.57	0.78	0.78	0.64	0.92
Conduct problems	0.39	0.31	0.48	0.33	0.25	0.40
Hyperactivity/Inattention	0.58	0.50	0.66	0.67	0.57	0.77
Peer relationship problems	**0.68**	**0.56**	**0.81**	**0.48**	**0.39**	**0.56**
Prosocial behavior	**0.98**	**0.80**	**1.17**	**0.60**	**0.49**	**0.71**

CI = Confidence Interval. Bold indicates statistically significant differences between genders, as evidenced by non-overlapping confidence intervals.

### Cross-gender predictive similarity

Based on the structural similarity model (best-fit model), sociodemographic, temperamental, and emotional variables were incorporated as predictors, to assess predictive similarity. Two models were tested for each set of predictors: one with predictors freely estimated across genders and another with predictors constrained to be equal across genders. As shown in Table 2, the models with gender-equal predictors were better supported for SES, temperament, and emotional variables, as indicated by lower BIC and SSABIC indices. This finding suggests that the predictors of the profiles are consistent across genders.

Table 4 presents the results of the MLR estimating the likelihood of early adolescent’s membership across distinct profiles. First, a multicollinearity test was conducted using variance inflation factors (VIF) to ensure that no two or more independent variables were highly correlated. All VIF values were below 2.5, indicating the absence of problematic multicollinearity^62^. Higher SES was associated with an increased likelihood of membership in the *Normative* profile compared to the other profiles. Higher effortful control predicted a lower probability of belonging to the *Moderate Externalizing* and *Comorbid* profiles relative to the *Normative* profile. In contrast, elevated surgency/extraversion was linked to a higher likelihood of membership in both the *Moderate Extern*alizing and *Comorbid* profiles, compared to the *Normative* profile. Surgency/extraversion also appears to differentiate the *Comorbid* and *Internalizing* profiles, showing a trend toward statistical significance (i.e., falling just short due to a narrow confidence interval margin of three hundredths). Negative affect did not significantly predict profile membership. Higher levels of emotion regulation increased the likelihood of belonging to the *Internalizing* profile relative to both the *Moderate Externalizing* and *Normative* profiles. Finally, greater lability/negativity was associated with an elevated probability of membership in the *Moderate Externalizing* and *Comorbid* profiles compared to the *Normative* profile. It also seems to be more related to *Internalizing* than to *Normative* profile with a tendency to significance.

**Table 4:** Multinomial Logistic Regression Results Examining the Influence of Predictors on Latent Profile Membership (Multigroup 4-Profile Structural Model).

Predictor	Moderately Externalizing vs Normative	Internalizing vs Normative	Comorbid vs Normative	Internalizing vs Moderately Externalizing	Comorbid vs Moderately Externalizing	Comorbid vs Internalizing
Socioeconomic status (T1)	**0.65 [0.46, 0.91]**	**0.42 [0.28, 0.64]**	**0.53 [0.33, 0.87]**	0.65 [0.34, 1.09]	0.83 [0.40, 1.69]	1.26 [0.59, 2.72]
Effortful control (T1)	**0.65 [0.48, 0.87]**	0.73 [0.51, 1.04]	**0.68 [0.47, 0.98]**	1.12 [0.65, 2.14]	1.04 [0.59, 1.84]	0.93 [0.50, 1.72]
Surgency/Extraversion (T1)	**1.32 [1.01, 1.73]**	0.88 [0.62, 1.24]	**1.51 [1.08, 2.12]**	0.66 [0.39, 1.07]	1.14 [0.69, 1.91]	1.73 [0.97, 3.08]
Negative affect (T1)	1.06 [0.81, 1.39]	1.04 [0.78, 1.38]	0.82 [0.56, 1.19]	0.97 [0.61, 1.68]	0.77 [0.45, 1.33]	0.79 [0.45, 1.38]
Emotion regulation (T2)	0.95 [0.55, 1.64]	**3.44 [1.33, 8.95]**	1.55 [0.59, 4.03]	**3.64 [1, 8.71]**	1.63 [0.44, 6.09]	0.45 [0.10, 2.25]
Lability/Negativity (T2)	**4.03 [1.86, 8.71]**	3.05 [0.97, 9.62]	**10.54 [3.94, 28.17]**	0.76 [0.15, 3.93]	2.61 [0.59, 11.59]	3.46 [0.57, 20.99]

Odds ratios are reported. Numbers in brackets represent 95% confidence intervals. Bold represent statistically significant results, as the interval does not include 1.

## Discussion

This study had two primary objectives: (1) to identify psychological maladjustment profiles in early adolescence based on gender and (2) to examine predictors from earlier developmental stages, specifically early and middle childhood. The analysis explored both distal, stable predictors (e.g., SES, temperament) and more proximal factors that are more adaptable and responsive to intervention (e.g., emotion regulation).

The initial step involved examining gender differences in the variables selected for profiling purposes. Consistent with previous literature on SDQ-SR scores, results from this preliminary comparison showed that boys in early adolescence exhibited higher levels of CP, hyperactivity/inattention and peer problems, whereas girls showed higher levels of emotional symptoms and prosocial behavior^63,64^. However, the observed effect sizes were small. When comparing boys and girls on temperamental variables, the findings partially aligned with existing research. Specifically, girls in early childhood scored higher on effortful control, while boys scored higher on surgency/extraversion^19,20^. Notably, girls also exhibited higher levels of negative affect. Previous research has often yielded inconsistent results or found no significant gender differences in this variable^19^. Recent reviews suggest that gender differences in negative affect, with girls scoring higher, may be more pronounced in early childhood compared to infancy or toddlerhood^65^. The relationship between gender and negative affect requires further exploration, particularly considering the different subdimensions of negative affect (e.g., emotionality sadness, difficultness, distress to limits, fear), which may exhibit varying patterns of gender differences^19,65^. Finally, girls in middle childhood scored higher on emotion regulation and lower on lability/negativity, aligning with the literature indicating that girls employ more adaptive emotion regulation strategies than boys, albeit with small effect sizes^28,66^.

Regarding the first objective of this study, which aimed to examine gender differences in psychological adjustment profiles among early adolescents, the results showed that both the number of profiles (i.e., four) and the mean scores of the compositional variables (i.e., emotional symptoms, CP, hyperactivity/inattention, peer relationship problems, and prosocial behavior) were similar across genders. However, differences were observed in the dispersion of the profiles (i.e., the variance of the compositional variables) and the distribution of girls and boys within these profiles.

Four distinct profiles were identified: *Moderately Externalizing*, *Internalizing*, *Comorbid*, and *Normativ*e. Previous research has highlighted various profiles of psychological adjustment among children and adolescents across diverse cultural contexts. For instance, studies in Spanish children and early adolescents, have identified three groups (e.g., externalizing, internalizing, and low risk^67^), while others have distinguished up to five profiles (e.g., high-difficulty, internalizing, externalizing, hyperactive, and well-adjusted^31^). In Chinese adolescents, three distinct risk categories have been identified (i.e., high-risk, middle-risk, and low-risk^37^). Unlike some previous studies^31^, our research did not identify a purely hyperactive group. Instead, high scores on hyperactivity/inattention were consistently linked to elevated scores on other externalizing variables, reinforcing the notion that these symptoms are part of the broader category of externalizing behaviors and are closely interconnected with other externalizing symptoms^68^. Moreover, while some studies^37^ have pinpointed quantitative categories (e.g., high, medium, and low risk), our study identified qualitatively distinct patterns, such as Internalizing and Moderately Externalizing. Thereby, as hypothesized, both internalizing and externalizing profiles, along with a co-occurring group, emerged, consistent with the distinctions commonly used in child and adolescent psychopathology^41^ and with the evidence that these problems often co-occur^6^. It should be noted that, following the broader SDQ structure^47^, peer problems were grouped with emotional symptoms under the internalizing dimension. As a result, both the *Internalizing* and the *Comorbid* profiles also capture difficulties in peer relations, suggesting that they may be better understood as reflecting broader psychosocial adjustment and highlighting the importance of peers in adolescent mental health.

In relation to differences in dispersion, greater variability was observed in certain variables depending on gender. The greater heterogeneity observed in boys’ prosocial behavior, compared to girls, might be influenced by gender stereotypes and social roles, which may encourage girls to conform more uniformly to caring and empathetic behaviors^69^. Girls who deviate from these expectations may face stronger social penalties^70^, which could lead to greater conformity and, consequently, less variability in their prosocial actions. In contrast, boys might experience more social flexibility and may be less likely to be penalized for not displaying prosociality, potentially resulting in a wider range of prosocial responses among boys. Finally, boys also exhibit greater variability in peer relationship problems, a pattern that might respond to the unique combination of social dynamics, behavioral tendencies, and peer group structures characteristic of adolescent boys. Research indicates that boys’ peer groups are often larger, more hierarchical, and centered around competition and dominance, which might lead to a wide range of peer experiences, from social acceptance and leadership to exclusion and conflict^71^.

Consistent with our expectations, girls tended to belong more to the *Internalizing* profile, while boys leaned towards the *Moderately Externalizing* profile. Our results also showed that girls were more likely to be in the *Normative* profile, and boys in the *Comorbid* profile. This aligns with previous literature on older adolescents indicating that girls tend to experience more internalizing and fewer externalizing problems than boys^10,11,36^. Our findings also support the notion that boys are more likely to be in co-occurring problem profiles^8^. The observed gender differences in rates of externalizing and internalizing problems during early adolescence can be attributed to multiple factors, including significant biological changes during puberty and increasing social pressures during adolescence^72^. These pressures may encourage boys to adopt more stereotypically masculine behaviors, such as assertiveness, while girls may be more inclined towards stereotypically feminine traits, such as empathy and sensitivity^73^. Consequently, boys may exhibit externalizing problems to mask underlying mental health issues, whereas girls may become more vulnerable to internalizing problems^29,74,75^. This highlights the importance of researchers and clinicians paying closer attention to gender-based differences in the onset and progression of mental health issues during early adolescence^76^.

The second aim of this study was to investigate the relationship between sociodemographic (i.e., SES), temperamental (i.e., effortful control, surgency/extraversion, and negative affect), and emotional (i.e., emotion regulation and lability) predictors and the profiles of psychological adjustment as a function of gender. The results showed that all predictors exhibited similar patterns across genders. Although some studies have suggested that certain temperamental factors (e.g., low effortful control and high negative affect) are associated with internalizing problems in girls and externalizing ones in boys^23^, other research has found no gender differences in the relationship between temperamental variables and externalizing problems^24,25^. Our results, based on a longitudinal perspective, with a rigorous multigroup analysis, endorse the latter perspective and show no significant gender differences in these associations. The discrepancies observed may be influenced by several factors. First, Rothbart et al.^23^ conducted a study examining the relationship between temperament and social behavior patterns in children aged 6–7 years. Part of the sample was studied cross-sectionally, while for a subset, some predictions were made based on infant observations. In contrast, our study tracks children across the transition from early childhood to early adolescence, a period characterized by significant developmental changes and new experiences. Furthermore, differences may arise from variations in the instruments used. For example, we employed the SDQ-SR, while Rothbart et al.^23^ used scales specifically tailored to their study. Additionally, our assessment of psychosocial adjustment problems relied on self-reports, whereas Rothbart et al.’s study was based on parental reports. Future research is needed to further investigate whether gender differences significantly influence the relationship between temperament and psychosocial adjustment problems, and whether this influence is consistent across informants and culture contexts.

As regards the effects of specific factors, our results indicated that high SES acted as a protective factor against problematic profiles when compared to the *Normative* profile; previous literature had also suggested that low SES is a risk factor for both internalizing^77^ and externalizing problems across genders^78^. Regarding temperamental predictors, effortful control emerged as a protective factor for problematic profiles, particularly those with high scores on externalizing problems (i.e., *Moderately Externalizing* and *Comorbid*), consistent with prior research^15^; thus, young children with low effortful control scores were 50% more likely to belong to the *Comorbid* profile compared to the *Normative* profile by early adolescence. Consistent with previous research^79,80^, early surgency/extraversion was identified as a risk factor for both externalizing and comorbid problems. However, our findings did not support the notion that extraversion serves as a protective factor against internalizing problems^81^. The discrepancy between the two studies may be attributable to methodological differences. Delgado et al.^81^ employed a cross-sectional design, focusing on early childhood (3–6 years), whereas the present study is longitudinal, tracking temperament in early childhood (4–7 years) and internalizing problems in early adolescence (10–12 years). It is possible that surgency/extraversion is a stronger predictor of internalizing problems in early childhood than in adolescence. Alternatively, unmeasured variables may be mediating or moderating the relationship between these constructs, warranting further investigation. Negative affect was not associated with any profile in this study, despite prior research associating it with both externalizing and internalizing problems^16,82^. Although multicollinearity tests (VIF < 2.5) indicated the absence of problematic multicollinearity among predictors, correlations suggest some conceptual overlap between negative affect and lability/negativity (*r* = 0.32–0.36; see Tables S1–S3). This overlap may help explain why negative affect did not emerge as a significant predictor. A previous study^81^ found that while negative affect independently influenced externalizing problems, its significance diminished when effortful control was considered, and no direct relationship was observed with internalizing problems. In our study, we hypothesize that the inclusion of emotional regulation variables may have further reduced the statistical significance of negative affect, suggesting the involvement of a posible mediating mechanism that has yet to be identified. Further research is needed to clarify the role of negative affect in conjunction with other temperamental traits.

The emotional regulation variables yielded seemingly paradoxical results. Specifically, high emotion regulation at middle childhood emerged as a risk factor for the *Internalizing* profile compared to the *Normative* and the *Moderately Externalizing* profiles. One possible explanation is that the *Internalizing* profile was mainly composed of girls, and although they tend to use adaptive emotion regulation strategies more frequently than boys, prior research suggests that this does not necessarily protect them from internalizing problems^83^. High emotion regulation might also indicate excessive control and behavioral restraint, contributing to internalizing problems in early adolescence^84^. Moreover, the ERC emotion regulation subscale includes socially valued behaviors for girls but not for boys, such as recognizing and expressing negative emotions^28^. However, excessive focus on overexpression of negative emotions may foster rumination, increasing vulnerability to internalizing problems^85^. These interpretations remain speculative and underscore the need for further research into how different facets of emotion regulation relate to internalizing problems^86^. In contrast, high lability/negativity emerged as a key risk factor for the *Moderately Externalizing*, *Internalizing* (with a trend toward significance), and *Comorbid* profiles, significantly increasing the likelihood of belonging to the *Comorbid* group by more than tenfold compared to the *Normative* group. From a longitudinal perspective, findings indicate that emotional lability during middle childhood/pre-adolescence serves as a strong predictor of developmental challenges in early adolescence^87^.

Notably, emotion regulation in middle childhood emerged as a more effective predictor of the *Internalizing* profile than temperamental variables observed in early childhood. In contrast, both early childhood and middle childhood variables served as predictors for externalizing profiles. This suggests that the *Internalizing* profile is more challenging to predict based on preschool measures, even when considering temperamental measures such as effortful control, surgency/extraversion, and negative affect. One possible explanation is that internalizing problems often manifest later and evolve more during adolescence, making these tendencies more covert and difficult to detect in early childhood^2^. Externalizing profiles, on the other hand, are easier to predict, possibly because they are more stable^88^ and deeply rooted in early, observable temperamental dispositions^89^.

### Limitations and strengths

This study has several limitations. Firstly, the attrition rate, typical of longitudinal studies, may have influenced the findings. The attrition rate was higher among participants from lower SES backgrounds, a common trend in longitudinal research^42^. This could introduce bias and limit the generalizability of the results to children with lower SES. Future studies should prioritize strategies to retain participants from disadvantaged backgrounds, as they are more likely to drop out of community-based longitudinal studies. Secondly, the sample was drawn from a specific geographic region (Galicia, Spain) which may restricted applicability of the findings to other populations or settings. Cultural context and characteristics of the regional educational system may have shaped the distribution of adjustment profiles observed. Finally, since it was necessary to fix the variances across profiles, as recommended when convergence issues are likely due to over-parameterization^59^, we were unable to determine with precision which profiles showed variance differences between boys and girls. Thus, we could only identify where differences emerged at the overall level.

Despite the limitations, this study offers an innovative approach by integrating developmental timing with gender-specific analysis, addressing inconsistencies in literature. In the field of psychosocial adjustment, multigroup analysis is central in advancing both theoretical and practical understanding. By formally evaluating whether the latent structure of adolescent psychological problems (e.g., profile shapes, sizes, or predictors) is equivalent across genders, it surpasses simple group comparisons. These insights contribute to a more comprehensive understanding of adolescent development and inform targeted strategies for improving psychosocial outcomes^40^. The study’s strengths also include the use of a longitudinal design with three time points, spanning five years (from 2018 to 2023), which facilitates the establishment of relationships between earlier variables at various stages of childhood and subsequent profiles. Furthermore, the inclusion of multiple informants at different time points (i.e., parents in early and middle childhood and children in early adolescence) helps mitigate informant bias (shared-method variance) in the predictions.

### Conclusion and implications

This three-wave longitudinal study examined whether psychological adjustment profiles, as measured by the SDQ-SR, exhibit similar patterns across genders in early adolescents. The analysis revealed four distinct profiles in both genders (i.e., *Moderately Externalizing*, *Internalizing*, *Comorbid*, and *Normative*). Although the means of the compositional variables were similar for boys and girls, differences emerged in the dispersion and distribution of their profiles. Boys showed greater heterogeneity in prosocial behavior and peer relationship problems. In terms of distribution, girls were more likely to belong to the *Internalizing* and *Normative* profiles, while boys were more often associated with the *Moderately Externalizing* and *Comorbid* profiles. The predictors of the profiles were consistent across genders. Low SES was associated with all problematic profiles. Effortful control, and surgency/extraversion were linked to the *Moderately Externalizing* and *Comorbid* profiles, and negative affect had no significant impact. Emotion regulation was specifically related to the *Internalizing* profile and lability/negativity appears to be a risk factor for all problematic profiles.

The results of this study hold both theoretical and practical significance. Theoretically, they underscore the importance of further exploring gender-specific factors to gain a deeper and more comprehensive understanding of psychological adjustment issues during early adolescence. Despite the absence of significant gender differences in profile predictors, future research should explore gender variations by incorporating additional variables, such as parenting practices, school, or peer variables, and testing in diverse sample types (e.g., clinical samples). Practically, this study highlights the potential to predict psychological maladjustment in early adolescence from an early age, emphasizing the importance of early risk identification and proactive intervention. Given adolescents’ heightened vulnerability to mental health challenges, timely and effective strategies are crucial^76^. Identifying distinct adjustment profiles offers valuable guidance for intervention. Adolescents with an internalizing profile may benefit from programs that foster adaptative emotion regulation, social skills, and coping, such as the FRIENDS program, a school-based cognitive–behavioral intervention with strong evidence for reducing anxiety and depression^90^. Those with a moderately externalizing profile may need support in behavioral regulation and impulse control, whereas the comorbid profile highlights the importance of multi-component approaches addressing behavior, emotion, and social competence. Even adolescents in the normative profile may benefit from universal preventive initiatives that strengthen protective factors and sustain adaptive functioning^91^. Focusing on temperamental traits as predictors from early childhood onward could enhance early intervention, as these traits are relatively stable and predictive of later maladjustment. The role of emotion regulation in internalizing problems also warrants careful attention, particularly regarding excessive or inadequate regulation. The observation that key predictors—such as SES, temperament, and emotion regulation—function similarly across genders supports their inclusion in early identification and intervention for both boys and girls. However, gender-related distinctions, such as differences in dispersion and profile distributions, highlight the need to consider gender in prevention efforts. Health-related programs should be adapted to be gender-sensitive, especially since girls, despite showing lower rates of externalizing and comorbid problems, are more likely to experience mental health issues in early and middle adolescence^92^.

## Supplementary Information

Below is the link to the electronic supplementary material.

Supplementary Material 1

## Data Availability

The datasets generated and analyzed during the current study are available from the corresponding author on reasonable request.

## References

[1] Solmi, M. et al. Age at onset of mental disorders worldwide: Large-scale meta-analysis of 192 epidemiological studies. *Mol. Psychiatry* **27**, 281–295 (2022). 34079068 10.1038/s41380-021-01161-7 PMC8960395

[2] World Health Organization. *World mental health report. Transforming mental health for all.* https://www.who.int/publications/i/item/9789240049338 (2022).

[3] Arslan, İB., Lucassen, N., Van Lier, P. A., De Haan, A. D. & Prinzie, P. Early childhood internalizing problems, externalizing problems and their co-occurrence and (mal) adaptive functioning in emerging adulthood: a 16-year follow-up study. *Soc. Psychiatry Psychiatr. Epidemiol.* **56**, 193–206 (2021). 32964254 10.1007/s00127-020-01959-w PMC7870752

[4] Bouter, D. C. et al. Five-year follow-up of the iBerry Study: screening in early adolescence to identify those at risk of psychopathology in emerging adulthood. *Eur. Child Adolesc. Psychiatry* **33**, 1–10 (2024). 38772966 10.1007/s00787-024-02462-2 PMC11618212

[5] Mulraney, M. et al. A systematic review of the persistence of childhood mental health problems into adulthood. *Neurosci. Biobehav. Rev.* **129**, 182–205 (2021). 34363845 10.1016/j.neubiorev.2021.07.030

[6] Álvarez-Voces, M., Díaz-Vázquez, B., López-Romero, L., Villar, P., & Romero, E. Gender differences in co-developmental trajectories of internalizing and externalizing problems: A seven-year longitudinal study from ages 3 to 12. *Child Psychiatry. Hum Dev.* (2024). 10.1007/s10578-024-01771-6 39425881

[7] Lawrence, T. I., Chen, L. & Kwok, O.-M. The distinct patterns and developmental comorbidity of adolescence depressive symptoms, impulsivity, and substance use. *J. Psychopathol. Behav. Assess.* **47**, 39 (2025).

[8] Shi, Q., Ettekal, I., Deutz, M. H. F. & Woltering, S. Trajectories of pure and co-occurring internalizing and externalizing problems from early childhood to adolescence: Associations with early childhood individual and contextual antecedents. *Dev. Psychol.* **56**, 1906–1918 (2020). 32816501 10.1037/dev0001095

[9] Zarakoviti, E., Shafran, R., Papadimitriou, D. & Bennett, S. D. The efficacy of parent training interventions for disruptive behavior disorders in treating untargeted comorbid internalizing symptoms in children and adolescents: a systematic review. *Clin. Child Fam. Psychol. Rev.* **24**, 542–552 (2021). 33991282 10.1007/s10567-021-00349-1 PMC8324591

[10] Campbell, O. L. K., Bann, D. & Patalay, P. The gender gap in adolescent mental health: A cross-national investigation of 566,829 adolescents across 73 countries. *SSM Popul Health.* **13**, 100742. 10.1016/j.ssmph.2021.100742 (2021). 33748389 10.1016/j.ssmph.2021.100742 PMC7960541

[11] Van Droogenbroeck, F., Spruyt, B. & Keppens, G. Gender differences in mental health problems among adolescents and the role of social support: results from the Belgian health interview surveys 2008 and 2013. *BMC Psychiatry* **18**, 6. 10.1186/s12888-018-1591-4 (2018). 29320999 10.1186/s12888-018-1591-4 PMC5763832

[12] Heaven, P. C. L., Newbury, K. & Mak, A. The impact of adolescent and parental characteristics on adolescent levels of delinquency and depression. *Pers. Indiv. Differ.* **36**, 173–185 (2004).

[13] Leve, L. D., Kim, H. K. & Pears, K. C. Childhood temperament and family environment as predictors of internalizing and externalizing trajectories from ages 5 to 17. *J. Abnorm. Child Psychol.* **33**, 505–520 (2005). 16195947 10.1007/s10802-005-6734-7 PMC1468033

[14] Shiner, R. L. Temperament and Personality in Childhood. In *Handbook of personality development* (eds Mroczek, D. K. & Little, T. D.) 213–230 (Lawrence Erlbaum Associates Publishers, 2006).

[15] King, K. M., Lengua, L. J. & Monahan, K. C. Individual differences in the development of self-regulation during pre-adolescence: Connections to context and adjustment. *J. Abnorm. Child Psychol.* **41**, 57–69 (2013). 22865096 10.1007/s10802-012-9665-0 PMC3529211

[16] Muris, P. & Ollendick, T. H. The role of temperament in the etiology of child psychopathology. *Clin. Child. Fam. Psychol. Rev.* **8**, 271–289 (2005). 16362256 10.1007/s10567-005-8809-y

[17] Putnam, S. P. & Rothbart, M. K. Development of short and very short forms of the children’s behavior questionnaire. *J. Pers. Assess.* **87**, 102–112 (2006). 16856791 10.1207/s15327752jpa8701_09

[18] Rothbart, M. K., Ahadi, S. A. & Evans, D. E. Temperament and personality: Origins and outcomes. *J. Pers. Soc. Psychol.* **78**, 122–135 (2000). 10653510 10.1037//0022-3514.78.1.122

[19] Else-Quest, N. M., Hyde, J. S., Goldsmith, H. H. & Van Hulle, C. A. Gender differences in temperament: A meta-analysis. *Psychol. Bull.* **132**, 33–72 (2006). 16435957 10.1037/0033-2909.132.1.33

[20] Smith, C. L. & Day, K. L. Parenting, anger, and effortful control as predictors of child externalizing behavior: The role of child sex as a moderator. *Int. J. Behav. Dev.* **42**, 248–256 (2018).

[21] Sanson, A., Hemphill, S. A., Yagmurlu, B. & McClowry, S. Temperament and social development. In *The Wiley-Blackwell Handbook of Childhood Social Development* (eds Smith, P. K. & Hart, C. H.) 227–245 (Wiley-Blackwell, 2011).

[22] Wang, F. L., Eisenberg, N., Valiente, C. & Spinrad, T. L. Role of temperament in early adolescent pure and co-occurring internalizing and externalizing problems using a bifactor model: Moderation by parenting and gender. *Dev. Psychopatol.* **28**, 1487–1504 (2016). 10.1017/S0954579415001224 PMC4900935 26646352

[23] Rothbart, M. K., Ahadi, S. A. & Hershey, K. L. Temperament and social behavior in childhood. *Merrill-Palmer Q.* **40**, 21–39 (1994).

[24] Olson, S. L., Sameroff, A. J., Kerr, D. C., Lopez, N. L. & Wellman, H. M. Developmental foundations of externalizing problems in young children: The role of effortful control. *Dev. Psychopathol.* **17**, 25–45 (2005). 15971758 10.1017/s0954579405050029

[25] Valiente, C. et al. The relations of effortful control and reactive control to children’s externalizing problems: A longitudinal assessment. *J. Pers.* **71**, 1171–1196 (2003). 14633062 10.1111/1467-6494.7106011

[26] Cicchetti, D., Ackerman, B. P. & Izard, C. E. Emotions and emotion regulation in developmental psychopathology. *Dev. Psychopathol.* **7**, 1–10 (1995).

[27] Aldao, A., Gee, D. G., De Los Reyes, A. & Seager, I. Emotion regulation as a transdiagnostic factor in the development of internalizing and externalizing psychopathology: Current and future directions. *Dev. Psychopathol.* **28**, 927–946 (2016). 27739387 10.1017/S0954579416000638

[28] Chaplin, T. M. & Aldao, A. Gender differences in emotion expression in children: A meta-analytic review. *Psychol. Bull.* **139**, 735–765 (2013). 23231534 10.1037/a0030737 PMC3597769

[29] Patel, V., Flisher, A. J., Hetrick, S. & McGorry, P. Mental health of young people: A global public-health challenge. *Lancet* **369**, 1302–1313 (2007). 17434406 10.1016/S0140-6736(07)60368-7

[30] Goodman, R. Psychometric properties of the strengths and difficulties questionnaire. *J. Am. Acad. Child. Adolesc. Psychiatry.* **40**, 1337–1345 (2001). 11699809 10.1097/00004583-200111000-00015

[31] Morales, A., Melero, S., Tomczyk, S., Espada, J. P. & Orgilés, M. Subtyping of strengths and difficulties in a Spanish children sample: A latent class analysis. *J. Affect. Disord.* **280**, 272–278 (2021). 33221712 10.1016/j.jad.2020.11.047

[32] Li, X. et al. Subtyping of internalizing and externalizing behaviors in Japanese community-based children: A latent class analysis and association with family activities. *Children* **9**, 210 (2022). 35204930 10.3390/children9020210 PMC8870000

[33] Patalay, P., Moulton, V., Goodman, A. & Ploubidis, G. B. Cross-domain symptom development typologies and their antecedents: Results from the UK millennium cohort study. *J. Am. Acad. Child. Adolesc. Psychiatry.* **56**, 765-776.e2 (2017). 28838581 10.1016/j.jaac.2017.06.009

[34] Rosato, N. C. & Baer, J. C. Latent class analysis: A method for capturing heterogeneity. *Soc. Work Res.* **36**, 61–69 (2012).

[35] Lanza, S. T. & Rhoades, B. L. Latent class analysis: An alternative perspective on subgroup analysis in prevention and treatment. *Prev. Sci.* **14**, 157–168 (2013). 21318625 10.1007/s11121-011-0201-1 PMC3173585

[36] Ortuño-Sierra, J., Fonseca-Pedrero, E., Sastre I Riba, S. & Muñiz, J. Patterns of behavioral and emotional difficulties through adolescence: the influence of prosocial skills. *An. Psicol.* **33**, 48–56 (2017).

[37] Ling, Y., Huebner, E. S., Yuan, H., Li, Z. & Liu, W. Subtyping of strengths and difficulties in a Chinese adolescent sample: A latent class analysis. *Child Indic. Res.* **9**, 933–948 (2016).

[38] Konrad, K. et al. Sex differences in psychiatric comorbidity and clinical presentation in youths with conduct disorder. *J. Child Psychol. Psychiatry Allied Discip.* **63**, 218–228 (2022). 10.1111/jcpp.13428 34008879

[39] Kam, C., Morin, A. J. S., Meyer, J. P. & Topolnytsky, L. Are commitment profiles stable and predictable? A latent transition analysis. *J. Manag.* **42**, 1462–1490 (2016).

[40] Morin, A. J. S., Meyer, J. P., Creusier, J. & Biétry, F. Multiple-group analysis of similarity in latent profile solutions. *Organ. Res. Methods.* **19**, 231–254 (2016).

[41] Achenbach, T. M., Ivanova, M. Y., Rescorla, L. A., Turner, L. V. & Althoff, R. R. Internalizing/externalizing problems: Review and recommendations for clinical and research applications. *J. Am. Acad. Child Adolesc. Psychiatry.* **55**, 647–656 (2016). 27453078 10.1016/j.jaac.2016.05.012

[42] Young, A. F., Powers, J. R. & Bell, S. L. Attrition in longitudinal studies: Who do you lose?. *ANZJPH.* **30**, 353–361 (2006). 10.1111/j.1467-842x.2006.tb00849.x 16956166

[43] American Psychiatric Association. *Diagnostic and Statistical Manual of Mental Disorders Fourth Edition Text Revision* (DSM-IV-TR). (American Psychiatric Association, 2000).

[44] Goodman, R., Ford, T., Simmons, H., Gatward, R. & Meltzer, H. Using the strengths and difficulties questionnaire (SDQ) to screen for child psychiatric disorders in a community sample. *Br. J. Psychiatry.* **177**, 534–539 (2000). 11102329 10.1192/bjp.177.6.534

[45] Ortuño-Sierra, J., Chocarro, E., Fonseca-Pedrero, E., Riba, S. S. I. & Muñiz, J. The assessment of emotional and behavioural problems: Internal structure of The strengths and difficulties questionnaire. *Int. J. Clin. Health Psychol.* **15**, 265–273 (2015). 30487843 10.1016/j.ijchp.2015.05.005 PMC6225034

[46] Keilow, M., Sievertsen, H. H., Niclasen, J. & Obel, C. The Strengths and Difficulties Questionnaire and standardized academic tests: Reliability across respondent type and age. *PLoS ONE* **14**, e0220193. 10.1371/journal.pone.0220193 (2019). 31344079 10.1371/journal.pone.0220193 PMC6657876

[47] Goodman, A., Lamping, D. L. & Ploubidis, G. B. When to use broader internalising and externalising subscales instead of the hypothesised five subscales on the Strengths and difficulties questionnaire (SDQ): Data from British parents, teachers and children. *J. Abnorm. Child. Psychol.* **38**, 1179–1191 (2010). 20623175 10.1007/s10802-010-9434-x

[48] López-Romero, L. et al. Testing the predictive and incremental validity of callous-unemotional traits versus the multidimensional psychopathy construct in preschool children. *J. Crim.* **80**, 101744 (2022).

[49] Shields, A., & Cicchetti, D. *Emotion Regulation Checklist* [Database record]. APA PsycTests (1995).

[50] Muthén, L. K., & Muthén, B. O. *Mplus User’s Guide* (7th ed.). (1998–2012).

[51] Enders, C. K. *Applied missing data analysis* (Guilford, 2010).

[52] Hickendorff, M., Edelsbrunner, P. A., McMullen, J., Schneider, M. & Trezise, K. Informative tools for characterizing individual differences in learning: Latent class, latent profile, and latent transition analysis. *Learn. Individ. Differ.* **66**, 4–15 (2018).

[53] McDermott, P. A., Rovine, M. J., Gerstner, C. C. E., Weiss, E. M. & Watkins, M. W. Latent profile analysis of classroom behavior problems in an American national sample of prekindergarten children. *Soc. Dev.* **31**, 1059–1078 (2022).

[54] Nylund, K. L., Asparouhov, T. & Muthén, B. O. Deciding on the number of classes in latent class analysis and growth mixture modeling: A Monte Carlo simulation study. *Struct. Equ. Model* **14**, 535–569 (2007).

[55] Marsh, H. W., Lüdtke, O., Trautwein, U. & Morin, A. J. S. Classical latent profile analysis of academic self-concept dimensions: Synergy of person- and variable-centered approaches to theoretical models of self-concept. *Struct. Equ. Model.* **16**, 191–225 (2009).

[56] Morin, A. J. S. et al. General growth mixture analysis of adolescents’ developmental trajectories of anxiety: The impact of untested invariance assumptions on substantive interpretations. *Struct. Equ. Model.* **18**, 613–648 (2011).

[57] Weller, B. E., Bowen, N. K. & Faubert, S. J. Latent class analysis: A guide to best practice. *J. Black Psychol.* **46**, 287–311 (2020).

[58] Litalien, D., Gillet, N., Gagné, M., Ratelle, C. F. & Morin, A. J. S. Self-determined motivation profiles among undergraduate students: A robust test of profile similarity as a function of gender and age. *Learn. Individ. Differ.* **70**, 39–52 (2019).

[59] Bauer, D. J. & Curran, P. J. The integration of continuous and discrete latent variable models: Potential problems and promising opportunities. *Psychol. Methods.* **9**, 3–29 (2004). 15053717 10.1037/1082-989X.9.1.3

[60] Morin, A. J. S. & Wang, J. C. A gentle introduction to mixture modeling using physical fitness performance data. In *An Introduction to Intermediate and Advanced Statistical Analyses for Sport and Exercise Scientists* (eds Ntoumanis, N. & Myers, N. D.) 183–209 (Wiley, London, 2016).

[61] Cohen, J. *Statistical Power Analysis for the Behavioral Sciences* (Routledge, 1988).

[62] Johnston, R., Jones, K. & Manley, D. Confounding and collinearity in regression analysis: A cautionary tale and an alternative procedure, illustrated by studies of British voting behaviour. *Qual. Quant.* **52**, 1957–1976 (2018). 29937587 10.1007/s11135-017-0584-6 PMC5993839

[63] Fonseca-Pedrero, E., Paino, M., Lemos-Giráldez, S., & Muñiz, J. Prevalencia de la sintomatología emocional y comportamental en adolescentes españoles a través del Strengths and Difficulties Questionnaire (SDQ). *Rev. Psicopatol. Psicol*. **16** (2011).

[64] Van Roy, B., Groholt, B., Heyerdahl, S. & Clench-Aas, J. Understanding discrepancies in parent-child reporting of emotional and behavioural problems: Effects of relational and socio-demographic factors. *BMC Psychiatry* **10**, 56. 10.1186/1471-244X-10-56 (2010). 20637090 10.1186/1471-244X-10-56 PMC2912799

[65] Putnam, S. P. et al. The Global Temperament Project: Parent-reported temperament in infants, toddlers, and children from 59 nations. *Dev. Psychol.* **60**, 916–941 (2024). 38573659 10.1037/dev0001732

[66] Orgilés, M., Morales, A., Fernández-Martínez, I., Ortigosa-Quiles, J. M. & Espada, J. P. Spanish adaptation and psychometric properties of the child version of the cognitive emotion regulation questionnaire. *PLoS ONE* **13**, e0201656. 10.1371/journal.pone.0201656 (2018). 30071082 10.1371/journal.pone.0201656 PMC6072061

[67] Fonseca-Pedrero, E., Ortuño-Sierra, J. & Pérez-Albéniz, A. Emotional and behavioural difficulties and prosocial behaviour in adolescents: A latent profile analysis. *Rev. Psiquiatría Salud Ment.* **13**, 202–212 (2020). 10.1016/j.rpsm.2020.01.002 32444209

[68] Waldman, I. D., Poore, H. E., van Hulle, C., Rathouz, P. J. & Lahey, B. B. External validity of a hierarchical dimensional model of child and adolescent psychopathology: Tests using confirmatory factor analyses and multivariate behavior genetic analyses. *J. Abnorm. Psychol.* **125**, 1053–1066 (2016). 27819467 10.1037/abn0000183 PMC6810679

[69] Eagly, A. H. The his and hers of prosocial behavior: An examination of the social psychology of gender. *Am. Psychol.* **64**, 644–658 (2009). 19899859 10.1037/0003-066X.64.8.644

[70] Meimoun, E., Aelenei, C. & Bonnot, V. Theorizing and studying reactions to gender norm violations. *Collabra Psychol.* **10**, 125682. 10.1525/collabra.125682 (2024).

[71] Rose, A. J. & Rudolph, K. D. A review of sex differences in peer relationship processes: Potential trade-offs for the emotional and behavioral development of girls and boys. *Psychol. Bull.* **132**, 98–131 (2006). 16435959 10.1037/0033-2909.132.1.98 PMC3160171

[72] Conley, C. S. & Rudolph, K. D. The emerging sex difference in adolescent depression: Interacting contributions of puberty and peer stress. *Dev Psychol.* **21**, 593–620 (2009). 10.1017/S0954579409000327 PMC2704098 19338700

[73] Wang, Y. N. Balanced authenticity predicts optimal well-being: Theoretical conceptualization and empirical development of the authenticity in relationships scale. *Pers. Individ. Differ.* **94**, 316–323 (2016).

[74] Anderson, A. S. et al. Youth coping and symptoms of anxiety and depression: Associations with age, gender, and peer stress. *Curr. Psychol.* **43**, 12421–12433 (2024). 40255870 10.1007/s12144-023-05363-w PMC12007690

[75] Gao, W., Ping, S. & Liu, X. Gender differences in depression, anxiety, and stress among college students: A longitudinal study from China. *J. Affect. Disord.* **263**, 292–300. 10.1016/j.jad.2019.11.121 (2020). 31818792 10.1016/j.jad.2019.11.121

[76] Scheiner, C., Grashoff, J., Kleindienst, N. & Buerger, A. Mental disorders at the beginning of adolescence: Prevalence estimates in a sample aged 11–14 years. *Public Health Pract.* **4**, 100348. 10.1016/j.puhip.2022.100348 (2022). 10.1016/j.puhip.2022.100348 PMC9761382 36545674

[77] Reiss, F. et al. Socioeconomic status, stressful life situations and mental health problems in children and adolescents: Results of the German BELLA cohort-study. *PLoS ONE* **14**, e0213700. 10.1371/journal.pone.0213700 (2019). 30865713 10.1371/journal.pone.0213700 PMC6415852

[78] Piotrowska, P. J., Stride, C. B., Croft, S. E. & Rowe, R. Socioeconomic status and antisocial behaviour among children and adolescents: A systematic review and meta-analysis. *Clin. Psychol. Rev.* **35**, 47–55 (2015). 25483561 10.1016/j.cpr.2014.11.003

[79] Derryberry, D. & Reed, M. A. Temperament and the self-organization of personality. *Dev. Psychopathol.* **6**, 653–676 (1994).

[80] Rothbart, M. K. & Putnam, S. P. Temperament and socialization. In *Paths to Successful Development: Personality in the Life Course* (eds Pulkkinen, L. & Caspi, A.) 19–45 (Cambridge University Press, 2002).

[81] Delgado, B., Carrasco, M. A., González-Peña, P. & Holgado-Tello, F. P. Temperament and behavioral problems in young children: The protective role of extraversion and effortful control. *J. Child Fam. Stud.* **27**, 3232–3240 (2018).

[82] Oldehinkel, A. J., Hartman, C. A., Ferdinand, R. F., Verhulst, F. C. & Ormel, J. Effortful control as modifier of the association between negative emotionality and adolescents’ mental health problems. *Dev. Psychopathol.* **19**, 523–539 (2007). 17459182 10.1017/S0954579407070253

[83] Nolen-Hoeksema, S. & Aldao, A. Gender and age differences in emotion regulation strategies and their relationship to depressive symptoms. *Pers. Indiv. Differ.* **51**, 704–708 (2011).

[84] Gilbert, K., Perino, M. T., Myers, M. J. & Sylvester, C. M. Overcontrol and neural response to errors in pediatric anxiety disorders. *J. Anxiety Disord.* **72**, 102224 (2020). 32289747 10.1016/j.janxdis.2020.102224 PMC7260107

[85] Nolen-Hoeksema, S., Wisco, B. E. & Lyubomirsky, S. Rethinking rumination. *Perspectioes Psychol. Sci.* **3**, 400–424 (2008). 10.1111/j.1745-6924.2008.00088.x 26158958

[86] Gross, J. J. Emotion regulation: Current status and future prospects. *Psychol. Inq.* **26**, 1–26 (2015).

[87] Kim-Spoon, J., Cicchetti, D. & Rogosch, F. A. A longitudinal study of emotion regulation, emotion lability-negativity, and internalizing symptomatology in maltreated and nonmaltreated children. *Child Dev.* **84**, 512–527 (2013). 23034132 10.1111/j.1467-8624.2012.01857.x PMC3794707

[88] Burt, K. B., Obradović, J., Long, J. D. & Masten, A. S. The interplay of social competence and psychopathology over 20 years: Testing transactional and cascade models. *Child Dev.* **79**, 359–374 (2008). 18366428 10.1111/j.1467-8624.2007.01130.x

[89] Schwartz, C. E., Snidman, N. & Kagan, J. Early childhood temperament as a determinant of externalizing behavior in adolescence. *Dev. Psychopathol.* **8**, 527–537 (2009).

[90] Filges, T., Smedslund, G., Eriksen, T. & Birkefoss, K. The FRIENDS preventive programme for reducing anxiety symptoms in children and adolescents: A systematic review. *Campbell Syst. Rev.* **19**, e1374 (2023). 38107252 10.1002/cl2.1374 PMC10723782

[91] Kim, B. K. E., Oesterle, S., Catalano, R. F. & Hawkins, J. D. Change in protective factors across adolescent development. *J. Appl. Dev. Psychol.* **40**, 26–37 (2015). 26405365 10.1016/j.appdev.2015.04.006 PMC4576918

[92] Yoon, Y., Eisenstadt, M., Lereya, S. T. & Deighton, J. Gender difference in the change of adolescents’ mental health and subjective wellbeing trajectories. *Eur. Child Adolesc. Psychiatry.* **32**, 1569–1578 (2023). 35246720 10.1007/s00787-022-01961-4 PMC8896070

