# Supplementary material for: Testing gender differences in psychological adjustment among early adolescents through a multigroup analysis of latent profiles
Source: Sci Rep. 2025 Nov 24;15:41547. doi: 10.1038/s41598-025-25420-7 (PMC12644576; doi:10.1038/s41598-025-25420-7)
Supplement: Supplementary file 1 — Supplementary Material 1 [file 41598_2025_25420_MOESM1_ESM.pdf]

Supplementary materials. Testing Gender Differences in Psychological Adjustment among Early Adolescents though a Multigroup Analysis of Latent Profiles

Table of Contents

Table S1. Correlations Among the Variables Studied in All Sample 3

Table S2. Correlations Among the Variables Studied in Boys 4

Table S3. Correlations Among the Variables Studied in Girls 5

Figure S1. Elbow plots of information criteria from latent profile analyses conducted separately for boys and girls 6

Table S4. Average Latent Class Probabilities for Most Likely Latent Class Pattern (Row) by Latent Class Pattern (Column) . 7

Table S5. Mean Estimates of the Multigroup 4-Profile Structural Similarity Across Profiles 8

Table S1

Correlations Among the Variables Studied in All Sample

Variable	1	2	3	4	5	6	7	8	9	10	11
1. Emotional symptoms (T3)											
2. Conduct problems (T3)	.29**										
3. Hyperactivity/Inattention (T3)	.27**	.54**									
4. Peer relationship problems (T3)	.41**	.37**	.26**								
5. Prosocial behavior (T3)	-.02	-.37**	-.26**	-.24**							
6. Socioeconomic status (T1)	-.16**	-.12**	-.09	-.14**	-.01						
7. Effortful control (T1)	0	-.17**	-.17**	-.10	.15**	-.08					
8. Surgency/Extraversion (T1)	-.05	.11*	.21**	.06	-.02	.08	-.02				
9. Negative affect (T1)	.13**	.02	0	-.01	.01	-.19**	.15**	-.03			
10. Emotion regulation (T2)	.05	-.10	-.06	-.04	.23***	.14**	.15**	.19**	-.13**		
11. Lability/Negativity (T2)	.12*	.26**	.26**	.12*	-.17**	-.15**	-.16**	.09	.32**	-.48**	

Note. T = Time. *$p < .005$; **$p < .001$. Bonferroni corrections applied to adjust for multiple comparisons.

Table S2

Correlations Among the Variables Studied in Boys

Variable	1	2	3	4	5	6	7	8	9	10	11
1. Emotional symptoms (T3)											
2. Conduct problems (T3)	.31**										
3. Hyperactivity/Inattention (T3)	.29**	.55**									
4. Peer relationship problems (T3)	.39**	.36**	.25**								
5. Prosocial behavior (T3)	-.05	-.39**	-.28**	-.22**							
6. Socioeconomic status (T1)	-.09	-.09	0	-.09	-.01						
7. Effortful control (T1)	-.10	-.11	-.16*	-.11	.01	-.04					
8. Surgency/Extraversion (T1)	-.10	.12	.19**	.07	-.01	.15	.01				
9. Negative affect (T1)	.15*	.03	.01	0	-.01	-.22**	.13	-.03			
10. Emotion regulation (T2)	.06	-.04	-.03	-.03	.18**	.11	.12	.21**	-.19*		
11. Lability/Negativity (T2)	.13	.22**	.27**	.15*	-.15	-.15	-.16*	.08	.36**	-.50**	

Note. T = Time. *p < .005; **p < .001. Bonferroni corrections applied to adjust for multiple comparisons.

Table S3

Correlations Among the Variables Studied in Girls

Variable	1	2	3	4	5	6	7	8	9	10	11
1. Emotional symptoms (T3)											
2. Conduct problems (T3)	.35**										
3. Hyperactivity/Inattention (T3)	.30**	.51**									
4. Peer relationship problems (T3)	.50**	.35**	.26**								
5. Prosocial behavior (T3)	-.06	-.27**	-.19**	-.23**							
6. Socioeconomic status (T1)	-.23**	-.18**	-.18**	-.20**	.01						
7. Effortful control (T1)	0	-.14	-.14	-.01	.23**	-.12					
8. Surgency/Extraversion (T1)	.01	.06	.22**	.03	.02	0	-.01				
9. Negative affect (T1)	.08	.06	.01	.02	-.02	-.16*	.12	-.01			
10. Emotion regulation (T2)	0	-.13	-.07	0	.24**	.18*	.12	.20**	-.09		
11. Lability/Negativity (T2)	.16*	.27**	.22**	.04	-.16*	-.17*	-.11	.09	.31**	-.41**	

Note. T = Time. **p* < .005; ***p* < .001. Bonferroni corrections applied to adjust for multiple comparisons.

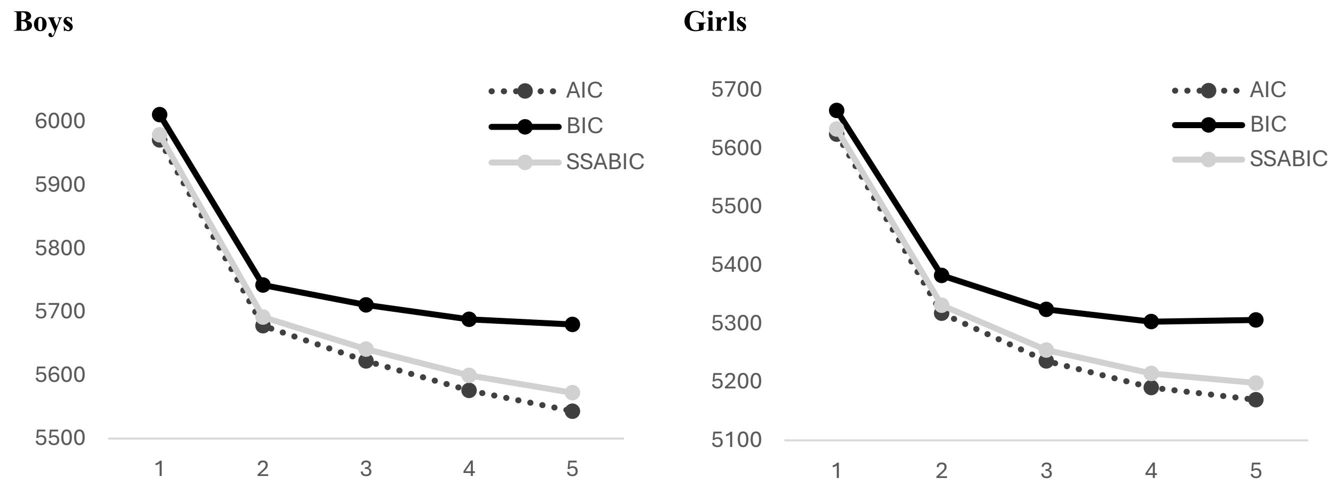

Figure S1. Elbow plots of information criteria from latent profile analyses conducted separately for boys and girls**.** the horizontal axis represents the number of profiles.

Table S4

Average Latent Class Probabilities for Most Likely Latent Class Pattern (Row) by Latent Class Pattern (Column)

	Profile 1: Moderately Externalizing	Profile 2: Internalizing	Profile 3: Comorbid	Profile 4: Normative
**Boys**				
Profile 1: Moderately Externalizing	.803	.032	.051	.113
Profile 2: Internalizing	.147	.751	.066	.036
Profile 3: Comorbid	.125	.025	.850	0
Profile 4: Normative	.082	.013	0	.905
**Girls**				
Profile 1: Moderately Externalizing	.792	.068	.008	.132
Profile 2: Internalizing	.057	.884	.018	.041
Profile 3: Comorbid	.085	.088	.827	0
Profile 4: Normative	.046	.013	0	.941

Note. Profiles were estimated using variables scores standardized as Z-scores, with a global mean of 0 and a global standard deviation of 1.

Table S5

Mean Estimates of the Multigroup 4-Profile Structural Similarity Across Profiles

Profile	Domain	Estimate	Lower CI	Upper CI
Profile 1: Moderately Externalizing	Emotional problems$_a$	-0.03	-0.24	0.17
	Conduct problems$_a$	0.64	0.36	0.93
	Hyperactivity/Inattention$_a$	0.66	0.50	0.82
	Peer relationship problems$_a$	-0.15	-0.29	-0.02
	Prosocial behavior$_a$	-0.23	-0.47	0.01
Profile 2: Internalizing	Emotional problems$_b$	1.40	1.11	1.70
	Conduct problems$_a$	0.38	0.01	0.75
	Hyperactivity/Inattention$_a$	0.36	0.07	0.65
	Peer relationship problems$_b$	1.51	0.96	2.06
	Prosocial behavior$_a$	-0.01	-0.25	0.22
Profile 3: Comorbid	Emotional problems$_c$	0.64	0.22	1.07
	Conduct problems$_b$	1.92	1.68	2.16
	Hyperactivity/Inattention$_b$	1.10	0.85	1.36
	Peer relationship problems$_b$	1.30	0.75	1.84
	Prosocial behavior$_b$	-1.30	-1.79	-0.80
Profile 4: Normative	Emotional problems$_a$	-0.32	-0.41	-0.23
	Conduct problems$_c$	-0.63	-0.70	-0.57
	Hyperactivity/Inattention$_c$	-0.52	-0.62	-0.41
	Peer relationship problems$_c$	-0.38	-0.46	-0.31
	Prosocial behavior$_c$	0.33	0.25	0.41

Note. Different subscript letters indicate statistically significant differences, based on non-overlapping confidence intervals. Lower CI = Lower 95% Confidence Interval; Upper CI = Upper 95% Confidence Interval.
